# Editorial: Epidemiology and clinical researches in atherosclerosis and cardiovascular disease

**DOI:** 10.3389/fcvm.2023.1212269

**Published:** 2023-05-16

**Authors:** Darui Gao, Yutong Samuel Cai, Yuesong Pan, Qian Ma, Wuxiang Xie

**Affiliations:** ^1^ Heart and Vascular Health Research Center, Peking University Clinical Research Institute, Peking University First Hospital, Beijing, China; ^2^ Key Laboratory of Epidemiology of Major Diseases (Peking University), Ministry of Education, Beijing, China; ^3^ Centre for Environmental Health and Sustainability, University of Leicester, Leicester, United Kingdom; ^4^ Department of Neurology, Beijing Tiantan Hospital, Capital Medical University, Beijing, China; ^5^ Department of Cardiology, Beijing Anzhen Hospital, Capital Medical University, Beijing, China

**Keywords:** atherosclerosis, cardiovascular disease, epimiology, cohort study, dementia

**Editorial on the Research Topic** Epidemiology and clinical researches in atherosclerosis and cardiovascular disease

Atherosclerosis is a systemic disease and the common cause of heart attacks, strokes and peripheral vascular disease collectively referred to as cardiovascular diseases (CVD), which are the leading cause of global mortality and a major contributor to disability and rising health care costs. Additionally, a wealth of epidemiological data demonstrated that atherosclerosis risk factors, including (but not limited to) hypertension, diabetes, and hyperlipidemia are associated with other chronic diseases such as chronic kidney disease, cognitive decline and dementia (1–6). The huge and still growing burden of CVD and dementia on individuals, families, and health-care systems indicates an urgent need for prevention and treatment measures on atherosclerotic diseases. Preventing severe atherosclerosis progression is expected to decrease high cardiovascular and dementia event rate.

However, there still exist challenges to be addressed. These challenges include but are not limited to (1) early detect participants with high-risk of CVD; (2) identify novel indicators for progression and prognosis of atherosclerotic diseases; (3) comorbidities of atherosclerotic diseases; (4) new drugs and therapies on atherosclerosis and CVD.

This research topic aimed at creating a forum for high-quality epidemiology and clinical researches in the field of atherosclerosis and CVD. The issue currently includes 12 papers on guiding comprehensive care and practice in preventing and managing major atherosclerotic CVD, including coronary heart disease, stroke, and peripheral vascular disease, and other chronic diseases which are associated with atherosclerosis.

In this topic, Wang et al. conducted a cohort study to explore the association between non-HDL-C and arterial stiffness on a large-scale Chinese population (https://www.frontiersin.org/articles/10.3389/fcvm.2022.981028/full). The results highlighted non-HDL-C as a potential risk factor for arterial stiffness, in especially for younger people. The clinical benefits of lowering non-HDL-C concentration should be further considered in the future.

Gao et al. performed a systematic review and meta-regression analysis to investigate the impact of statins on CRP/hsCRP reduction on coronary plaque burden measured using total atheroma volume (TAV), percent atheroma volume (PAV), and plaque volume (PV) (https://www.frontiersin.org/articles/10.3389/fcvm.2022.989527/full). After adjusting for percent change of LDL-C, age, gender and study duration, this meta-regression analysis mainly found that the percent change of CRP/hsCRP was significantly associated with the change of TAV/PV. The results indicated that statins promote plaque regression, which may be associated to their capacity to reduce inflammation.

In a multi-ethnic longitudinal cohort study, Anbar et al. compared carotid atherosclerosis in Europeans (EA), South Asian (SA), and African Caribbean (AC) participants in the Southall and Brent Revisited (SABRE) study and they found that the prevalence of any plaque was comparable in EA and SA, although it was lower in AC. Total plaque area, numbers of plaques, plaque class, or greyscale median did not differ by ethnicity in individuals who had plaque (https://www.frontiersin.org/articles/10.3389/fcvm.2022.1002820/full). This study indicated that the similarity of plaque burden in SA and EA despite established differences in atherosclerotic CVD risk casts some doubt on the utility of carotid ultrasound as a means of assessing risk across these ethnic groups.

Sundquist et al. performed a population-based follow-up study to examine the role of mtDNA-CN in heart failure (HF) incidence and its role in the association between myocardial infarction (MI) and HF. In addition, this study also investigated the role of mtDNA-CN in overall and HF mortality (https://www.frontiersin.org/articles/10.3389/fcvm.2022.1012403/full). This study mainly found that low baseline mtDNA-CN is a molecular risk factor for HF incidence and may be a risk factor for overall and HF-related mortality.

In a cohort study published in this topic (https://www.frontiersin.org/articles/10.3389/fcvm.2022.1026597/full), Moon et al. examined the association between height loss and the prevalence of CVD using data from a sizable Korean cohort. The participants were divided into three groups based on their annual height loss: Group 1 (height loss: <0.3 cm/year), Group 2 (height loss: 0.3 to <0.6 cm/year), or Group 3 (height loss: ≥0.6 cm/year). The results indicated that the incidence of major adverse cardiac and cerebral event was substantially higher in Groups 2 and 3 than in Group 1. In the Korean population, the severity of height reduction was independently correlated with the occurrence of CVD.

Muhammad et al. conducted a longitudinal two-cohort analysis, and identified association between positive triglyceride-glucose (TyG) index and increased arterial stiffness and increased incidence of diabetes, CE, stroke, and all-cause and cardiovascular mortality (https://www.frontiersin.org/articles/10.3389/fcvm.2022.1035105/full). The results of this work represent preliminary evidence that TyG index can potentially be helpful in the identification of those at increased long-term risk of adverse health outcomes.

A classification tree analysis (CTA) model established by Ruan et al. in this topic identified four key correlates of depressive disorders: loneliness was the most salient, followed by arthritis, family relationship, and heart disease (https://www.frontiersin.org/articles/10.3389/fcvm.2022.1035203/full). Due to the potential for modification or treatment, these findings regarding the four main correlates of depressive disorders are clinically interesting. The clinical needs for collaborative multidisciplinary management services—which integrate social work outreach services to foster family relationships, mental health services to relive loneliness, and primary care services to manage arthritis and heart disease—are further indicated by the significant interactions between the four major factors.

In a cohort study, Ma et al. recruited 299 patients with new-onset non-valvular atrial fibrillation (AF) between 2013 and 2015 at the Department of Cardiovascular Medicine of the Southwest Hospital of the Army Medical University (Third Military Medical University) in Chongqing, China (https://www.frontiersin.org/articles/10.3389/fcvm.2022.1072164/full). The findings revealed that throughout the median follow-up period of 28 (IQR: 27, 29) months, IL-34 and IL-38 were independently associated with stroke and all-cause mortality in patients with AF. Additionally, IL-38 and NT-proBNP considerably increased the CHA2DS2-VASc score's capacity to predict AF-related all-cause death.

In another large-scale cohort study, Hua et al. found that participants with and without heart disease experienced similar changes in global cognitive scores during the pre-pandemic period, however, in comparison to the group without heart disease, the heart disease group experienced a greater decline in the global cognitive score during the pandemic period (https://www.frontiersin.org/articles/10.3389/fcvm.2022.1077800/full). The findings highlight the need for rapid cognitive monitoring and therapies for the population suffering from heart diseases.

Grabitz et al. focused on exploring the early indicators and rivers of cardiovascular disease in young athletes pursuing a career in competitive sports (https://www.frontiersin.org/articles/10.3389/fcvm.2023.1081675/abstract). They discovered an unexpectedly high rate of cardiovascular risk factors despite regular exercise and the absence of obesity. Their findings suggested that children and young adults, who initially appeared to be in good condition, require rigorous medical examinations. To further investigate potential negative impacts on vascular health, long-term monitoring of those who began engaging in excessive physical activity as children and young seems required.

In this topic, Ni et al. employed linkage disequilibrium score (LDSC) regression and a two-sample Mendelian randomization (MR) framework to systematically examine the causal interplay between key factors that influence vascular calcification and CVD, as well as longevity (https://www.frontiersin.org/articles/10.3389/fcvm.2023.1096662/full). The results provide evidence for a causal relationship between VK1 levels and CVD risk as well as a genetic genetic correlation between serum Ca and VD and CVD risk. Cardiovascular risk can be decreased by maintaining appropriate serum Ca (2.376 mmol/L) and VD levels (46.8 nmol/L).

In the last article published in this topic, Wright et al. examined associations between a history of pregnancy loss and incident CVD among participants in the Women's Health Initiative Observational Study (https://www.frontiersin.org/articles/10.3389/fcvm.2023.1108286/full). In this cohort study of postmenopausal women aged 50–79, history of stillbirth was strongly associated with a risk of cardiovascular outcomes within 5 years of baseline. Additionally, history of pregnancy loss, and of stillbirth, may be a therapeutically effective marker of cardiovascular disease risk in women.

In conclusion, the articles published in this research topic provide additional evidence from epidemiology and clinical researches for current literature on atherosclerosis and cardiovascular disease. Nevertheless, incredible challenges on the prevention and treatment of atherosclerosis and cardiovascular disease need more attention following the aging of the population and the development of social economy. We thank the authors for their cutting-edge works, and also express our gratitude to all the reviewers for their generously devoted time and highly valuable comments. Finally, we hope that the reader will enjoy these articles.
